# Adoptive Cell Therapy with Tumor-Infiltrating Lymphocytes in Advanced Melanoma Patients

**DOI:** 10.1155/2018/3530148

**Published:** 2018-03-19

**Authors:** Mélanie Saint-Jean, Anne-Chantal Knol, Christelle Volteau, Gaëlle Quéreux, Lucie Peuvrel, Anabelle Brocard, Marie-Christine Pandolfino, Soraya Saiagh, Jean-Michel Nguyen, Christophe Bedane, Nicole Basset-Seguin, Amir Khammari, Brigitte Dréno

**Affiliations:** ^1^Dermato-cancerology Department, CHU Nantes, Place Alexis Ricordeau, 44093 Nantes, France; ^2^CIC1413, CRCINA INSERM U1232, CHU Nantes, Place Alexis Ricordeau, Nantes, France; ^3^Research Leading Department, CHU Nantes, Place Alexis Ricordeau, 44093 Nantes, France; ^4^Cell and Gene Therapy Unit, CHU Nantes, Place Alexis Ricordeau, 44093 Nantes, France; ^5^SEME, CIC1413, CRCINA INSERM U1232, CHU Nantes, Place Alexis Ricordeau, Nantes, France; ^6^Dermatology Department, University Hospital, 2 avenue Martin Luther King, 87042 Limoges Cedex, France; ^7^Dermatology Department, Saint-Louis Hospital, 1 avenue Claude-Vellefaux, 75475 Paris Cedex 10, France

## Abstract

Immunotherapy for melanoma includes adoptive cell therapy with autologous tumor-infiltrating lymphocytes (TILs). This monocenter retrospective study was undertaken to evaluate the efficacy and safety of this treatment of patients with advanced melanoma. All advanced melanoma patients treated with TILs using the same TIL expansion methodology and same treatment interleukin-2 (IL-2) regimen between 2009 and 2012 were included. After sterile intralesional excision of a cutaneous or subcutaneous metastasis, TILs were produced according to a previously described method and then infused into the patient who also received a complementary subcutaneous IL-2 regimen. Nine women and 1 man were treated for unresectable stage IIIC (*n* = 4) or IV (*n* = 6) melanoma. All but 1 patient with unresectable stage III melanoma (1st line) had received at least 2 previous treatments, including anti-CTLA-4 antibody for 4. The number of TILs infused ranged from 0.23 × 10^9^ to 22.9 × 10^9^. Regarding safety, no serious adverse effect was reported. Therapeutic responses included a complete remission, a partial remission, 2 stabilizations, and 6 progressions. Among these 4 patients with clinical benefit, 1 is still alive with 9 years of follow-up and 1 died from another cause after 8 years of follow-up. Notably, patients treated with high percentages of CD4 + CD25 + CD127lowFoxp3+ T cells among their TILs had significantly shorter OS. The therapeutic effect of combining TILs with new immunotherapies needs further investigation.

## 1. Introduction

The potential interest of immunotherapy for melanoma is based on the finding of early spontaneous regressions of primary melanomas [1] or cutaneous metastases and, even more rarely, metastatic locations. Such regression seems to be related to immunological mechanisms, particularly through the expression of some cytokines in the tumor microenvironment and via autoimmune factors (observation of vitiligo and halo nevi parallel to tumor regression).

The immunotherapeutic strategy for melanoma consists of either authorized approaches such as checkpoint inhibitors, cytokine administration (interleukin-2 (IL-2) or interferon) or experimental treatments including adoptive T cell therapy (ACT) with tumor-infiltrating lymphocytes (TILs) [2], and active vaccination. Over the last few decades, metastatic melanoma treatment has been revolutionized by 2 new active immunotherapy classes: anticytotoxic T-lymphocyte antigen-4 (CTLA-4) and antiprogrammed death-1 receptor (PD-1) antibodies. Notably, ipilimumab-treated metastatic melanoma patients had significantly longer overall survival (OS) than those treated with gp100 vaccination [3] or combined with dacarbazine versus dacarbazine alone [4]. In parallel, it was shown that nivolumab and pembrolizumab significantly improved the prognoses of these patients [5, 6].

ACT with TILs was first developed by Rosenberg's team in 1988. Indeed, at the end of the 1980s, it was shown that the TILs in melanoma can be grown in the presence of IL-2 and that they recognize autologous tumor cells [7]. That finding served as the basis of ACT for melanoma that obtained a 34% objective response rate in 86 melanoma patients treated with TILs and high-dose IL-2 [8]. Several later studies included patient conditioning before TIL infusion employing nonmyeloablative chemotherapy with or without total body irradiation. Lymphodepletion was shown to increase the response rate to around 50% [9, 10] and to improve the durability of response at several independent centers [11–14].

In Nantes, we developed this approach using TILs derived from patients in an adjuvant setting after lymph node excision for regional metastatic melanoma (American Joint Committee on Cancer (AJCC) stage III). In a randomized study comparing TILs (without lymphodepletion) and IL-2 versus IL-2 alone, for the subgroup of patients with 1 invaded lymph node, survival without relapse was longer for those that received the combined regimen [15–17]. Those results were confirmed in a recent long-term update [18].

In our hospital, TILs are prepared by the dedicated Cell and Gene Therapy Unit and our group has acquired solid experience in ACT in the adjuvant setting. In addition, TILs can be generated rapidly (<1 month) and the technique is easily reproducible (95% success). That explains why we proposed this therapeutic approach on a compassionate basis to 10 patients with advanced melanoma at a therapeutic impasse. Unlike our team's previous studies that concerned patients in an adjuvant setting, this is the first study on advanced melanoma patients. The objectives of this study were to evaluate ACT efficacy and safety as last-line treatment in advanced melanoma patients.

## 2. Materials and Methods

### 2.1. Patient Selection, Treatment, and Follow-Up

This retrospective monocenter study included all the patients treated with TILs and IL-2 for advanced melanoma between February 2009 and June 2012. All the patients had histologically proven metastatic melanoma with at least 1 not-entirely-resectable cutaneous or subcutaneous metastasis available for sampling to generate TILs, with a least 2 previous treatments that failed including 1 chemotherapy line. No concomitant treatment for melanoma was allowed (chemotherapy, targeted or radiation therapy). All the patients were informed and gave their consent to receive this treatment. The French Agence Nationale de Sécurité du Médicament Agency (National Agency for Drug Safety) provided an exceptional measure authorization for each patient in view of the therapeutic impasse.

Unlike previously published TIL studies, the patients did not receive a preconditioning lymphodepleting regimen neither high-dose IL-2. Indeed, our previous study of adoptive transfer of melanoma-specific cytotoxic T lymphocyte clones in metastatic melanoma patients demonstrated that clinical responses were associated with the expansion of additional melanoma-specific T cells [19]. This suggested that adoptive therapy may help to recruit other melanoma-specific T cells, phenomenon also demonstrated in vaccination studies. This intermolecular or intramolecular epitope spreading could only occur in absence of preconditioning regimen in fully immunocompetent patients. A cutaneous or subcutaneous metastasis was partially excised under sterile conditions in the operating room by a trained surgical team. No minimal size criterion for tumor excision was needed. Five weeks later, the polyclonal TILs were infused into the patient. IL-2 (Proleukin, Chiron, Emeryville, CA, USA) subcutaneous administration (6 × 10^6^ IU/day 5 days/week for 2 weeks) was started the day of TIL infusion. A second TIL infusion followed by IL-2 injections, according to the same scheme, was administered 1 month later. Patients' monthly evaluations included a complete physical examination with measurement of skin targets and blood and biochemical laboratory analyses every 15 days during the first 2 months then every 2 months. The radiological response to therapy according to RECIST criteria was evaluated every 3 months [20]. Patients with a complete response (CR) and partial response (PR) were defined as “responders.” Patients with stable disease (SD) longer than 6 months were defined with CR and PR patients as “patients with clinical benefit.” The date and location of recurrence and date of death were recorded. Adverse events were noted using Common Terminology Criteria for Adverse Events v4.0 [13].

### 2.2. Data Collected

The following information was recorded for each patient: sex; age; primary melanoma: date of diagnosis, localization, Breslow index (in mm); any lymph node excision with the number of invaded nodes; *BRAF*, *NRAS*, and c-*KIT* mutational status; concerning metastatic disease: the date of its diagnosis, AJCC stage (unresectable III or IV) and location(s) of metastases, blood lactate dehydrogenase (LDH) level at metastasis diagnosis, systemic treatments before TIL treatment with dates, types of treatment(s) and responses; details of the treatment with TILs, that is, performance status (PS) during treatment, biopsy site used to produce the TILs, number of TIL infusions received, number of TILs received, percentages of tumor-specific TILs, therapeutic response, tolerance, and disease-free and overall survival times; treatment(s) received after the TILs with date(s), type(s), and response(s); and, finally, if applicable, date of death or date of latest updates. The censoring date for the statistical analysis was 1 October 2014. For long-term survivors, an update was obtained on 1 January 2018.

### 2.3. TIL Production from Cutaneous or Subcutaneous Metastasis

TILs were cultured in Good Manufacturing Practice conditions in the Cell and Gene Therapy Unit (University Hospital, Nantes, France) according to a previously described procedure [21, 22]. Briefly, short-term cultured TILs were isolated by culturing fragments of cutaneous metastases into 12-well tissue culture plates with X-VIVO 15 serum-free medium (BioWhittaker, Walkersville, MD, USA) containing 150 U/ml recombinant interleukin-2 (rIL-2) (Eurocetus, Rueil-Malmaison, France) and glutamine (1 mM, BioWhittaker) for 10–14 days. Ex vivo expanded TILs were derived as follows: 1.8 × 10^6^ short-term cultured TILs were plated at 300 viable lymphocytes/well with irradiated feeder cells (allogeneic peripheral blood leukocytes (PBL) and B-EBV cells: Epstein-Barr virus-infected B-cells) into U-bottom microplates in 150 *μ*l of rIL-2 medium. PHA-L (phytohemagglutinin-L or leucoagglutinin) (Difco, Detroit, ML, USA) was added on day 0 (1 *μ*g/ml). Ten days later, lymphocytes were recovered from the culture plates, adjusted to 1 × 10^6^ cells/mL in rIL-2 medium, and transferred into culture cell bags for an additional 10 days. The final TIL harvest was obtained by centrifuging, washing, and suspending the TILs in 4% human serum albumin (LFB, Les Ulis, France). Starting from cryopreserved, short-term cultured TILs, a second TIL expansion was obtained within 1 month of the first. Aliquots of the generated TILs to be infused into the patient were cryopreserved for subsequent analysis of each patient's tumor specificity once the autologous tumor cell line had been established in culture. Melanoma cell lines were established as previously described [23, 24] and were successfully established for 6 tumor samples [24]. RNA extraction, reverse transcription, PCR, and gene expression analyses were performed on frozen tumor tissue samples as previously described [25].

### 2.4. Characterization of TILs

#### 2.4.1. Cytokine Production Assay to Evaluate the Percentages of Tumor-Specific TILs

The fraction of tumor-reactive TILs was determined from flow cytometry identification of interferon-gamma- (IFN-*γ*-) secreting T cells among TILs stimulated by the autologous melanoma cell line, as described previously [22], and according to the method described by Jung et al. [26].

#### 2.4.2. Antibodies and Flow Cytometry Analyses

The following antibodies were used: PE anti-CD2 (clone S5.2), PE anti-CD56 (clone MY31), and PE anti-CD25 (clone 2A3), all from BD Pharmingen (Le Pont de Claix, France). We also used PC5 anti-CD3 (clone UCHT1), PE anti-CD8 (clone B9.11), APC anti-CD4 (clone 13B8.2), APC anti-CD19 (clone J3-119), PC7 anti-CD45 (clone J.33), and PE anti-CD16 (clone 3G8), all from Beckman Coulter (Marseille, France). To evaluate regulatory T cell- (Treg-) associated markers, we used 5-color multiparametric analysis with the following antibodies: FITC anti-CD4 (clone RPA-T4), PE anti-CD127 (clone hIL-7R-M21), PE-Cy7 anti-CD25 (clone M-A251), BD V450 Horizon anti-CD3 (clone UCHT1), all from Becton Dickinson, and APC anti-Foxp3 (clone 236A/E7) from eBioscience (San Diego, CA, USA). Lymphocytes were gated according to their forward- and size-scatter characteristics, and FACSCanto analyses used the BDFACS Diva software (BD Biosciences, San Jose, CA, USA).

### 2.5. Statistical Analyses

Wilcoxon and Fisher's exact tests were used to compare survivors' or patients with clinical benefit results to those of deceased patients or with progressive disease. The relationship between survival and each parameter was assessed using Spearman's correlation test. OS was defined as the time elapsed from the date of the first TIL infusion to that of death from any cause. The response duration lasted from the date of the first TIL infusion to that of the first recurrence or progression. A Cox proportional hazards model was used to assess the predictive value of continuous parameters. R statistical software was used and statistical significance was set at *P* < 0.05.

## 3. Results

### 3.1. Patients

Ten patients (9 women and 1 man) were treated with TILs (Table 1). Their primary melanomas were located on a lower limb for 7, the back for 2, and on the scalp of 1. Mean Breslow index was 3.06 (range 0.74–5.75) mm. All but 1 patient had previously received at least 2 other treatments, including chemotherapy for 8, with dacarbazine, carboplatin, temozolomide, vindesine, fotemustine, or cyclophosphamide; vaccination for 4; anti-CTLA4 antibody ipilimumab for 2 (10 or 3 mg/kg for 1 each); NCT00324155 protocol: dacarbazine (850 mg/m^2^ every 3 weeks) + ipilimumab (10 mg/kg every 3 weeks) for 2; NCT00338130 protocol comparing AstraZeneca AZD6244 versus temozolomide, radiotherapy delivered to a cutaneous metastasis on the lower limb, and surgery for small intestine resection. Only patient 6, a 76-year-old woman with an unresectable acral melanoma had received no prior treatment. Five patients had wild-type *BRAF* status, 4 had the V600E mutation, and the status of 1 was unknown (patient referred from another center). None of the patients had previously received a BRAF inhibitor. Indeed, vemurafenib was accorded marketing authorization in February 2012 in France; however, the 3 patients treated with ACT in this study in 2012 were wild-type *BRAF* carriers. Patient 7 had a c-*KIT* mutation.

### 3.2. TIL Treatment

TILs were successfully expanded for all the patients (Table 2). At the time of TIL infusion, patients were 17–88 (mean 62) years old. The mean time between metastatic disease diagnosis and TIL infusion was 3.6 years (range 1 month–10.75 years). For the 4 patients treated with ipilimumab before TILs, the mean interval was 16 months. Four patients were AJCC stage IIIC and 6 were stage IV with cutaneous (*n* = 9/10) lymph node (3/10), lung (3/10), liver (3/10), digestive (2/10), bone (1/10), and adrenal (1/10) metastases. The PS score was at 0 for 7 patients, 1 for 2, and 2 for 1. LDH levels before TIL infusion were normal for half the patient and above the upper normal limit for the other half. A subcutaneous nodule or cutaneous metastasis was used to generate TILs for 4 and 6 patients, respectively. Notably, patient 4 had only 1 cutaneous metastasis with a diameter larger than 8 cm, which was intralesionally removed to produce TILs. In this particular case, the surgery was aimed to reduce tumor burden but patient #4 was not rendered disease-free by surgery. The number of infused TILs ranged from 0.23 to 22.9 × 10^9^ (mean 7.1 × 10^9^) per infusion (2 infusions/patient except for patient 2 who received 4). No serious adverse event was noted (1 patient suffered grade 3 nausea and vomiting). The observed adverse events were described previously and are known to be attributable to IL-2 and not TILs: asthenia for 6, anorexia for 3, myalgias for 2, nausea for 2, and, for 1 each thrombocytopenia, vomiting, ageusia, pruritus, cutaneous rash, flu-like syndrome, or chills.

Therapeutic responses after TIL treatment were: patient 4's CR, patient 2's PR, and patients 8 and 10's SD, considered with clinical benefit, and 6 progressions deemed without clinical benefit. Patient 2's PR lasted 8 months. Patient 8's SD was confirmed and lasted 17 months. Evaluated at 3 months, patient 10 had SD, confirmed at 6 months, and PD at 9 months. No significant differences were found for therapeutic responses to TILs or OS according to clinical characteristics (Tables 3 and 4).

### 3.3. Treatments Received after TILs (Table 5)

After TIL cycles, patient 4 (CR) and patient 8 (palliative care because of major progression with poor general condition) received no further treatment. Patients 5, 7, and 10 received ipilimumab that achieved 2 PRs and 1 SD; patient 2 received MEK (mitogen-activated protein kinase kinase) inhibitor that led to SD. Finally, chemotherapy was prescribed as follows: fotemustine alone for patient 3, dacarbazine combined with fotemustine for patient 6, or carboplatin for patients 9 and 10, and achieved, respectively, 1 PD, 1 CR, 1 PD, and 1 SD. Eight patients died, 2 patients are still alive with median follow-up at 8.8 (range 8.4–9.2) years. Among these 2 survivors, patient 2 had responded to ACT, while patient 5 was an ACT nonresponder but achieved a CR after receiving ipilimumab twice. Notably, patient 4 died recently from another cause than melanoma and was still on melanoma CR after ACT with a follow-up of more than 8 years.

### 3.4. Characterization of Melanoma Cells

RT-PCR analysis was possible for 7 of the 10 patients (samples not available for the other 3). Melanoma antigens, including Melan-A, tyrosinase, and gp-100, were strongly expressed at the RNA level. On the contrary, only patient 3′ melanoma cells expressed NY-ESO1 (New York esophageal squamous cell carcinoma 1) RNA (details not shown). No association was found between melanoma-antigen expression and the response to TILs or OS (Table 4).

### 3.5. Characterization of TILs

#### 3.5.1. TIL Phenotypes

Fluorescence-activated cell sorting (FACS) analyses were conducted to evaluate TIL characteristics. The generated TILs comprised high percentages of CD3+ lymphocytes (range 90.3–100%) that coexpressed CD8 (range 7.1–82.1%) or CD4+ T cells (range 10.4–93.5%). Among the CD4 + CD25+ T cell population, we isolated a subgroup of CD127lowFoxp3+ T cells (range 2.7–34.6%) that represented 1.51% of the total CD3+ population on average (range 0.07–7.57%). Notably, OS was significantly shorter for (*P* < 0.05, Table 4) patients with higher percentages of CD4 + CD25 + CD127lowFoxp3+ T cells among their TILs.

#### 3.5.2. Evaluation of the Percentages of Tumor-Specific TILs

The percentage of interferon-*γ*-producing TILs in response to autologous melanoma cell line stimulation could be evaluated for 5 patients; it ranged from 0.15% to 5.23% (Table 6). The other 5 patients' cell lines derived from skin nodules had been contaminated with bacteria.

## 4. Discussion

Herein, we presented the results of ACT with TILs for 10 advanced melanoma patients at a therapeutic impasse. ACT obtained clinical benefit in 4 patients (including 2 patients with objective responses) receiving third-line or beyond therapy, with CR in patient 4, PR in patient 2, and patients 8 and 10 had SD. Patients 2 and 10, who were first responders, experienced secondary escape after a median of 7.5 months. The objective response rate in this study is 20% lower than the ones reported in other ACT studies using lymphodepletion from 27.5% [11] to 50% [9]. In our study, TILs were successfully expanded in all the cases and the 10 patients received the complete treatment course as planned; whereas there are frequent patient dropouts in other ACT trials that can be due to rapid progressive disease, no TIL expansion or lymphodepleting conditioning regimen-related adverse events [27]. When comparing the objective response rates on intention-to-treat basis, the one from this study of 20% is near the other ones reported from 18% [12] to 37% [9] but with a better tolerance.

Notably, 4 patients received ipilimumab before TILs: TIL-responding patients 2 and 8, and nonresponding patients 1 and 3. After TIL therapy, ipilimumab was given to 3 patients who responded: patient 5: PR then SD; patient 7: early PR then progression; and patient 10: SD. Receiving ipilimumab before or after TIL therapy was not significantly associated with a better therapeutic response or longer OS.

Even if we included patients in third-line or more melanoma treatment, this study was conducted before the revolution in treatment options for metastatic melanoma, notably anti-PD-1 antibody. The efficacy of TILs alone at an advanced disease stage is limited because of the immunodeficient microenvironment. New treatments, such as immune-checkpoint inhibitors, could counter this local immunodeficiency, thereby justifying a therapeutic strategy combining TILs with them. Our team previously published very encouraging results when combining TILs with intralesional administration of adenovirus expressing interferon-*γ* [28].

As now well-described with immunotherapy, we have 3 long-term responders. Indeed, among the 10 ACT-treated patients, patient 2 and 5 are still alive, patient 4 died from another cause and was still on melanoma CR with median follow-up exceeding 8 years. The survivors include 1 ACT responder that had several treatments after ACT, including a MEK inhibitor. The 2nd survivor did not respond to ACT but achieved CR after 2 cycles of ipilimumab, suggesting the potential benefit of combining ACT and ipilimumab.

Concerning ACT toxicity, no grade 4 side effect was reported; only grade 3 nausea and vomiting was noted. All other adverse events were grade 1 or 2 and were linked to IL-2 injections and not to TILs. Based on our results, this approach has a very acceptable toxicity profile, including in elderly patients (patient 8 was 88 years old at TIL infusion). Moreover, we did not observe any increase of a previously experienced toxicity, for example with ipilimumab, which is a highly relevant finding when combined treatments are given.

Our approach has several originalities compared to previously reported studies. First, unlike all the other teams using ACT with TILs, our patients were not conditioned with lymphocyte-depleting chemotherapies or total body irradiation [10]. Indeed, we previously showed that, in the adjuvant setting, TIL efficacy was observed without such conditioning [15]. Bypassing conditioning enabled us to shorten the duration of hospitalization and avoid infectious complications. In this study, the mean duration of the hospitalization was 2 days for all the patients, with 24-hour clinical monitoring after TIL infusion. In the other studies, hospitalization lasted 19.8 [9] to 23 days [29] because of serious adverse events, such as thrombocytopenia requiring platelet transfusion (median 30 units) [9] and febrile neutropenia (affecting 50–100% of the patients [9, 29]).

Moreover, low-dose IL-2 was used to limit toxicity without reducing its efficacy on lymphocyte activation. The feasibility of ACT using subcutaneous low-dose IL-2, instead of high-dose intravenous IL-2, was investigated in a 2012 pilot study by Ellebaek et al. [12]. Those authors demonstrated that complete and durable responses were obtained after combined ACT and low-dose IL-2 (2 MIU/day for 2 weeks) with significantly less toxicity. Our results confirmed that finding, with the same regimen than the one used in the adjuvant setting (6 MIU/day, 5 days per week for 2 weeks).

Our choice to use low doses of IL-2 is aimed to favor the persistence of infused T cells and avoid the expansion of Tregs in patients, which could be deleterious for the efficiency of ACT. Indeed, IL-2's key role favoring transferred T cell survival has previously been reported by Yee et al. [30]. We can raise the question of a potential direct antitumor effect of IL-2. However, we used a dose 4 times lower than the therapeutic high-dose intravenous IL-2. Moreover, in the adjuvant setting, we previously showed that IL-2 alone had a lower efficacy compared to combined TILs and IL-2 [16].

Interestingly, according to our results, OS was significantly shorter for ACT-treated patients with higher percentages of CD4 + CD25 + CD127lowFoxp3+ T cells among their TILs. Because TILs are expanded from the T cell population harbored in the cutaneous or subcutaneous metastasis, we could hypothesize that patients with poorer prognoses had larger Treg populations in their lesions. However, our team previously showed that the subpopulation of CD4 + CD25+ T lymphocytes including Tregs decreased during the TIL-generation culture but at an earlier stage of the disease [31]. Finally, another possibility could be that the CD4 + Foxp3+ T lymphocytes among infused TILs might be activated T lymphocytes with transiently upregulated Foxp3 expression [32]. Notably, another study including 5 ACT trials found that levels of peripheral CD4 + Foxp3+ Tregs were negatively associated with clinical response to adoptive immunotherapy in melanoma patients [32]. The absence of an examination of CD4 + CD25 + CD127lowFoxp3+ T cell functionality is a limitation of our study.

## 5. Conclusion

In summary, our results showed that ACT without a lymphocyte-depleting regimen and with subcutaneous low-dose IL-2 was safe in heavily pretreated advanced melanoma patients. A higher percentage of CD4 + CD25 + CD127lowFoxp3+ T cells among the infused TIL population was associated with significantly shorter OS. Although therapeutic responses to ACT are rare at advanced disease stages, as shown herein with a small number of patients having objective responses, ACT remains a pertinent therapeutic alternative. The combination of ACT with checkpoint inhibitors could potentiate the TIL effect by countering local immunodeficiency and warrants further investigation.

## Figures and Tables

**Table 1:** Advanced melanoma patients' characteristics before ACT.

Patient	Sex	At diagnosis			Advanced or metastatic disease		Treatments before TILs			*BRAF*	c-*KIT*
		Localization	Breslow (mm)	AJCC Stage	Age	Metastasis diagnosis to TIL injection (years)	Chemotherapy	Anti-CTLA4 (ipilimumab to TILs time, months)	Others	Status	Status
1	F	Scalp	3	II	15	1.6	Fote	Ipilimumab [16]	RT, Surg	WT	WT
2	F	Left ankle	2	NA	36	1.6	DCB	BMS^a^ [17]		V600E	WT
3	F	Back	5	II	68	2.4	DCB, carbo, cycloP	BMS^a^ [27]		V600E	WT
4	F	Back	5.2	II	59	5.3	DCB, carbo, vind, temo		Vac	V600E	WT
5	F	Left leg	1.21	NA	38	2.5	DCB, carbo, paclitaxel		RT	NA	WT
6	F	Left foot	2.85	II	76	0.1	None	None	None	V600E	WT
7	F	Left leg	0.74	I	62	10.1	DCB, carbo		Vac, AZD	WT	Mutated
8	M	Right foot	5.75	II	87	0.8	DCB, temo	Ipilimumab [5]	Vac	WT	WT
9	F	Left leg	1.85	I	82	0.9	DCB			WT	WT
10	F	Right leg	3	II	63	10.75			Vac	WT	WT

AJCC: American Joint Committee on Cancer; AZD: NCT00338130 study (AstraZeneca AZD 6244 versus temo); BMS: study (dacarbazine + ipilimumab 10 mg/kg versus dacarbazine + placebo); carbo: carboplatin; cycloP: cyclophosphamide; DCB: dacarbazine; Fote: fotemustine; NA: data not available; RT: radiotherapy; Surg: surgery; temo: temozolomide; Vac: vaccination; vind: vindesine; WT: wild type. ^a^Patient in the dacarbazine + ipi arm.

**Table 2:** Patients' characteristics during ACT.

Patient	Sex	Age	PS	LDH level	AJCC stage	Biopsy site used for TIL extraction	Size of excised tumor (cm^3^)	TIL infusion		TIL amount injected (10^9^)	Metastases	Response	Adverse events		PFS (months)	OS (months)
								Year	*n*				Grade 1-2	Grade 3		
1	F	17	0	2 N	IV	SCnod	6	2009	2	8.9	Skin, liver, lung, adrenal, small intestine	PD	Asthenia, anorexia, thrombocytopenia			12.9
2	F	37	0	1.5 N	IV	SCnod	1	2009	4	13.35	Skin, LN, lung	PR then PD	Asthenia, myalgias	Nausea, vomiting		110^a^
3	F	71	0	2 N	IV	SCnod	3	2009	2	9.0	Skin	PD			3.0	5.7
4	F	64	0	N	IIIC	Cnod	147	2010	2	1.87	Skin	CR				100
5	F	41	0	N	IV	SCnod	5	2010	2	0.23	SC, lung, liver, colon	PD	Asthenia, myalgia, nausea		3.3	100^a^
6	F	76	0	1.5 N	IV	Cnod	36	2011	2	4.70	Skin	PD			2.0	14.8
7	F	72	1	N	IV	Cnod	6	2011	2	22.90	Skin, LN, bone	PD	Anorexia, rash		3.0	12.3
8	M	88	2	1.5 N	IIIC	Cnod	2	2012	2	4.20	Skin	SD	Ageusia, asthenia, pruritus			31.8
9	F	83	1	N	IIIC	Cnod	140	2012	2	3.10	Skin, LN	PD	Asthenia, flu-like syndrome		4.9	8.4
10	F	73	0	N	IIIC	Cnod	0.75	2012	2	3	Skin	SD then PD	Anorexia, asthenia, flu-like syndrome		7.9	45.0

AJCC: American Joint Committee on Cancer; Cnod: cutaneous nodule; CR: complete response; Skin: cutaneous; N: normal range; PD: progressive disease; PFS: progression-free survival; PR: partial response; PS: performance status; SD: stable disease; SC: subcutaneous; SCnod: subcutaneous nodule. ^a^Ongoing.

**Table 3:** Comparisons of characteristics between patients with clinical benefit (CR, PR, and SD) and patients without clinical benefit (PD).

Variable	*N*	Patients with clinical benefit (*n* = 6)	Patients without clinical benefit (*n* = 4)	*P* value
Clinical data				
Breslow index, median (mm)	10	2.44	3.99	0.134
PS > 0	10	2/6	1/4	1.000
LDH > N	10	3/6	2/4	1.0000
AJCC stage IV	10	5/6	1/4	0.191
Metastatic disease diagnosis to TIL injection, median (years)	10	2.9	4.6	0.669
Ipilimumab before TILs	10	2/6	2/4	1.000
Ipilimumab after TILs	10	2/6	1/4	1.000
TILs infused, median (×10^9^)	10	8.1	5.5	0.609
*BRAF* mutation V600E	9	2/5	2/4	1.000
c-*KIT* mutated	10	1/6	0/4	1.000
TIL phenotypes				
CD3+	9	97.70%	96.23%	0.914
CD3 + CD4+	9	56.98%	47.04%	0.914
CD3 + CD8+	9	34.58%	36.78%	0.914
CD4 + CD25+^a^	9	8.50%	5.56%	1.000
CD4 + CD25 + CD127lowCTLA4+	9	16.89%	19.07%	0.3524
CD4 + CD25 + CD127lowFoxp3+^b^	9	30.71%	20.49%	0.1714

AJCC: American Joint Committee on Cancer; CI: confidence interval; LDH: lactate dehydrogenase; PS: performance status. ^a^Percentage among the CD3+ population. ^b^Percentage among the CD4 + CD25+ population.

**Table 4:** Univariate analysis of overall survival.

Variable	*n*	OR [95% CI]	*P* value
Clinical data			
Breslow index (mm)	10	1.13 [0.7514–1.718]	0.545
PS > 0	10	3.448 [0.67–17.6]	0.137
LDH > N	10	1.3 [0.32–5.4]	0.716
AJCC stage IV	10	0.81 [0.198–3.28]	0.763
Metastatic disease diagnosis to TIL infusion (years)	10	1.01 [0.835–1.23]	0.887
Ipilimumab before TILs	10	1.1 [0.26–4.57]	0.913
Ipilimumab after TILs	10	0.59 [0.12–2.98]	0.526
TILs infused (×10^9^)	10	1.04 [0.94–1.16]	0.449
*BRAF* mutation V600E	9	0.38 [0.07–2.03]	0.260
c-*KIT* mutated	10	3.97 [0.36–43.9]	0.261
Melanoma cell PCR			
MAGE-1	7	0.41 [0.04–4.68]	0.476
MAGE-3	7	1.04 [0.11–9.66]	0.503
Melan-A	7	17.5 [0.21–1483]	0.207
NY-ESO-1	6	NA	0.999
Na17A	7	9.58 [0.22–411]	0.239
gp100	7	0.86 [0.04–16.8]	0.923
Tyrosinase	7	NA	0.998
TIL phenotypes			
CD3+	9	NA	0.643
CD3 + CD4+	9	3.15 [0.28–35.6]	0.355
CD3 + CD8+	9	4.78 [0.14–166]	0.388
CD4 + CD25+	9	NA	0.071
CD4 + CD25 + CD127lowCTLA4+	9	0.97 [0.02–57.97]	0.987
CD4 + CD25 + CD127lowFoxp3+	9	16E7 [43.4–6E12]	**0.011** ^∗^

AJCC: American Joint Committee on Cancer; CI: confidence interval; LDH: lactate dehydrogenase; MAGE-1 and -3: melanoma antigen-1 and -3; NA: data not available; NY-ESO1: New York esophageal squamous cell carcinoma 1; PS: performance status. ^∗^Significant value.

**Table 5:** Other treatments received after ACT.

Patient	Other treatments received after TILs					
	First	Response	Second	Response	Third	Response
1	NA	NA				
2	Lilly study (tasisulam versus paclitaxel)	NA (study suspended)	Fote	NA	MEKi	SD
3	Fote	PD				
4	None					
5	Ipilimumab	PR	Ipilimumab	SD	Surg (single skin met)	CR
6	Fote DCB	CR				
7	Ipilimumab	PR at 2 mo then PD				
8	None					
9	Carbo DCB	PD				
10	Carbo DCB	SD	Ipilimumab	SD		

Carbo: carboplatin; CR: complete response; DCB: dacarbazine; Fote: fotemustine; MEKi: MEK inhibitor; met: metastasis; mo: months; NA: data not available; PD: progressive disease; PR: partial response; SD: stable disease; Surg: surgery.

**Table 6:** Proportion of specific tumor-TILs.

Patient	TILs CD8^+^ IFN-*γ*^+^	
	R1 (%)	R2 (%)
1	1.23	5.23
4	NA	3.2
5	NA	1.13
6	0.17	0.15
7	1.08	1.41

R1: first infusion of TILs; R2: second infusion of TILs; IFN-*γ*^+^: interferon-gamma; NA: data not available.

## Abbreviations

ACT: Adoptive cell therapy
AJCC: American Joint Committee on Cancer
CR: Complete response
CTLA-4: Cytotoxic T-lymphocyte antigen-4
MEK: Mitogen-activated protein kinase
PD: Progressive disease
PD-1: Programmed death-1 receptor
PFS: Progression-free survival
PR: Partial response
PS: Performance status
SD: Stable disease
TILs: Tumor-infiltrating lymphocytes
Tregs: Regulatory T lymphocytes.
